# Molecular recognition and induced dimerization of hnRNP A2/B1 truncations by G-quadruplex single strand DNA

**DOI:** 10.1038/s41598-026-44646-7

**Published:** 2026-03-26

**Authors:** Dilidaer Shahatibieke, Xiaohui Tang, Xuanfang Zheng, Yue Liu, Abudoureyimu Abula

**Affiliations:** 1 Department of microbiology, School of Basic Medical Sciences, Xinjiang Medical University, Urumqi, China; 2 Xinjiang Key Laboratory of Molecular Biology for Endemic Diseases, Xinjiang Medical University, Urumqi, China; 3 State Key Laboratory of Pathogenesis, Prevention, Treatment of Central Asian High Incidence Diseases, Xinjiang Medical University, Urumqi, China; 4 The State Key Laboratory of Pharmaceutical Biotechnology, School of Life Sciences, Nanjing University, Nanjing, China

**Keywords:** HnRNP A2/B1 Truncations, Induced dimerization, G-quadruplex ssDNA, Biochemistry, Biotechnology, Molecular biology

## Abstract

Heterogeneous nuclear ribonucleoprotein A2/B1 (hnRNP A2/B1) is a multifunctional nucleic acid metabolism regulator with established roles in viral infection and tumorigenesis. However, a critical gap exists between the oligomeric state of hnRNP A2/B1 and its function as a nuclear DNA sensor. To address this gap, we generated full-length hnRNP A2/B1 and three domain-truncated variants (△NLS, RRM-PrLD, RRM-RGG) using SUMO/MBP fusion expression systems. To define the nucleic acid binding and oligomeric properties of these variants, we combined SEC with EMSA and ITC. These analyses revealed that full-length hnRNP A2/B1 forms soluble amorphous aggregates, whereas the truncated variants exist as stable homogeneous monomers under in vitro solution conditions. Additionally, results demonstrated that the RRM-RGG (15–250) variant binds to ssDNA but not dsDNA. Notably, SEC combined with CD and AUC confirmed that RRM-RGG (15–250) truncations undergo homodimerization induced by 12nt and 22nt Guanine quadruplex (G4) structure enriched ssDNA, which is abundant in the genomes of diverse viruses. Structure predictions revealed that the C-terminal PrLD, and NLS domain are intrinsically disordered, a feature potentially underlying the protein’s aggregation propensity and crystallization recalcitrance. NPDock simulations demonstrated that G4-structured ssDNA binds and stabilizes the RRM-RGG (15–250) truncation via non-conserved key residues that are distinct from those of other hnRNP family members. This work provides a biophysical basis for hypothesizing that G4-structured ssDNA-dependent dimerization may contribute to the protein’s antiviral function, and establishes a biophysical framework to guide future investigations into the protein’s antiviral mechanism and the design of rational targeted inhibitors.

**Supplementary Information:**

The online version contains supplementary material available at 10.1038/s41598-026-44646-7.

## Introduction

In gene expression regulation and disease pathogenesis research, nucleic acid-binding proteins remain frontier targets in life sciences^1,2^. Heterogeneous nuclear ribonucleic protein A2/B1 (hnRNP A2/B1), an abundant, functionally critical hnRNP family member^3^, localizes to the nucleus and is a research focus due to its roles in nucleic acid metabolism and pathology^4–7^. Studies have demonstrated that hnRNP A2/B1 generates two major isoforms—hnRNP A2 and hnRNP B1—via alternative splicing. The A2 isoform consists of 341 amino acids, while the B1 isoform possesses a unique structure of 353 amino acids, resulting from the insertion of 12 amino acid residues at its N-terminus^8^. The N-terminus of hnRNP A2/B1 comprises two tandem RNA recognition motifs (RRMs), an RGG domain enriched in arginine-glycine-glycine (RGG) repeats, along with a C-terminal prion-like domain (PrLD) and an M9 nuclear localization signal (PY-NLS) domain^9^. The RRM domains enable the precise recognition and binding of target RNAs in core processes, including RNA transcriptional regulation, splicing processing, nucleocytoplasmic transport, and stability maintenance^10^. In contrast, the RGG domain not only participates in nucleic acid interactions through positively charged residues but also plays an irreplaceable role in mediating the assembly of protein-protein complexes and driving liquid-liquid phase separation^11^.

The diverse domain architecture of hnRNP A2/B1 endows it with a complex network of biological functions. Current research indicates that hnRNP A2/B1 acts not only as a core regulator of RNA metabolism but also is deeply involved in multiple pathophysiological processes, such as viral infection responses, tumorigenesis and progression, and neurological disorders^12–14^. A particularly groundbreaking discovery is the identification of hnRNP A2/B1 as a novel pathogen DNA sensor within the host cell nucleus, which provides a fresh perspective for deciphering the molecular mechanisms of antiviral immunity^15^. Upon stimulation by HSV-1 viral DNA, hnRNP A2/B1 undergoes homodimerization, binds to the demethylase Jumonji Domain-Containing Protein 6 to complete demethylation modification, and is subsequently translocated to the cytoplasm to activate the TBK1-IRF3 signaling pathway. This ultimately induces the production of type I interferons, establishing a host innate immune barrier^15^. In our previous investigations, we proposed a novel hypothesis positing that hnRNP A2/B1 may regulate its oligomeric states by binding to U-shaped DNA structures, thereby exerting its antiviral functions^16^. U-shaped DNA refers to a broad category of nucleic acid conformations characterized by a folded, hairpin-like topology with an acute bend of approximately eighty degrees^17^. Guanine quadruplex (G4) structures are a subtype of U-shaped DNA, formed by the stacking of G-quartets via Hoogsteen hydrogen bonds^18^. Distinct from other U-shaped motifs (DNA hairpins with Watson-Crick base pairing), G4 structures are stabilized by G-quartet stacking and require a minimum of four guanine-rich tracts, a feature that renders them abundant in viral genomes and oncogene promoters^19–21^. Studies have shown that hnRNP A2/B1 and its homologous proteins play key roles in regulating viral replication, telomere homeostasis, and the expression of tumor-associated genes^22^.

Despite significant advancements in functional studies of hnRNP A2/B1, research on the biophysiological and structural biology of its full-length form remains bottlenecked. To date, only the crystal structure of the nucleic acid-binding domain truncate RRMs (15–193) of hnRNP A2/B1 in complex with 12nt single-stranded RNA (ssRNA) has been resolved, revealing the molecular mechanism underlying its preferential recognition of ssRNA sequences^23^. Homologous hnRNP family members, such as hnRNP A1 and hnRNP D, have been shown to bind nucleic acids and form 1:1 complex. For instance, hnRNP A1 forms a 1:1 complex to regulate mRNA splicing, whereas hnRNP D also forms a 1:1 complex via its RGG domain to modulate telomere homeostasis^23–26^. However, the oligomeric states and diverse nucleic acid-binding properties of hnRNP A2/B1—distinct from its homologs in domain architecture—remain poorly characterized, highlighting the need for systematic, specific DNA-binding biophysical studies.

In our prior research, we resolved the crystal structure of the RRMs (15–193) truncate in complex with 12nt G4 ssDNA and elucidated the molecular mechanisms governing its recognition of U-shaped ssDNA structures and unique dimerization^16^. Despite advances in understanding hnRNP A2/B1’s functional roles, three critical questions remain unresolved: (1) What are the oligomeric states of full-length hnRNP A2/B1 and its domain-truncated variants in vitro? (2) What is the reason for hnRNP A2/B1’s aggregation and crystallization challenges? (3) Which kind of DNA binding induce hnRNP A2/B1 dimerization, and what is the binding properties underlying this interaction? To address these gaps, we combined biophysical characterization techniques—size-exclusion chromatography (SEC), analytical ultracentrifugation (AUC), isothermal titration calorimetry (ITC), and electrophoretic mobility shift assay (EMSA)—with structural prediction to systematically investigate the oligomeric regulation and G4 ssDNA dependent dimerization of hnRNP A2/B1, thereby providing mechanistic insights into its biological functions.

Our findings reveal that full-length hnRNP A2/B1 with fusion tags exhibited amorphous aggregation, whereas three tag-cleaved truncates (△NLS (1–313), RRM-PrLD (15–293), and RRM-RGG (15–250)) remained stably monomeric in vitro solution. Notably, the RRM-RGG (15–250) truncate displayed selective binding to ssDNA and underwent G4-structure induced homodimerization. Structure prediction showed that ~ 75% of hnRNP A2/B1’s N and C-termini are intrinsically disordered, potentially underlying their aggregation and crystallization challenges. Our findings clarify the oligomeric states of full-length hnRNP A2/B1 and its truncated variants, provide a biophysical basis for hypothesizing that G4-structured ssDNA-dependent dimerization may contribute to the protein’s antiviral function, and establish a biophysical framework to guide future investigations into the protein’s antiviral mechanism and the rational design of targeted inhibitors.

## Methods

### Construction of expression vectors

The hnRNP A2/B1 gene (Gene Bank ID: 3181) and its truncated variant were synthesized and amplified via polymerase chain reaction (PCR), then cloned into pET28a-SUMO and pMAT9s-MBP expression vectors via homologous recombination. The gene was inserted between Ulp1/BamH1 sites in pET28a-SUMO, and SARS-Mpro/BamH1 sites in pMAT9s-MBP. Recombinant plasmids were transformed into DH5α cells, and positive clones were selected and verified by sanger sequencing.

### Protein expression and purification

Recombinant plasmids were transformed into *Escherichia coli* Rosetta (DE3) competent cells and inoculated into TB medium with kanamycin and chloramphenicol. Cultures were grown at 37 °C, 200 rpm until OD600 reached 0.6–0.8, then induced with 0.2 mM isopropyl-β-D-thiogalactopyranoside (IPTG) at 16 °C, 200 rpm for 20 h. After cell harvesting, pellets were resuspended in lysis buffer (50 mM Tris-HCl, pH 8.0, 150 mM NaCl, 500 µL 100 mM PMSF) and disrupted at 1400 bar. Lysates were centrifuged at 4 °C, 18,000 rpm for 40 min, supernatants filtered (0.22-µm) and purified using an AKTA Avant system. For His-Sumo tagged proteins, a His Trap HP 5 ml (GE Healthcare) Ni²⁺-affinity column was equilibrated with Solution A1 (50 mM Tris-HCl, pH 8.0; 500 mM NaCl; 5% glycerol) and eluted with 300 mM imidazole. MBP-tagged proteins were purified using an MBP Trap HP 5 ml (GE Healthcare) column equilibrated with Solution A2 (50 mM Tris-HCl, pH 8.0; 300 mM NaCl) and eluted with 10 mM maltose. Preliminarily purified proteins were digested with Ulp1 or SARS-Mpro proteases at 4 °C overnight, followed by a second Ni²⁺-affinity chromatography step. Finally, the purity and molecular weight of the purified target proteins were comprehensively evaluated using sodium dodecyl sulfate-polyacrylamide gel electrophoresis (SDS-PAGE) analysis.

### Size exclusion chromatography (SEC)​

**Fig. 1:**
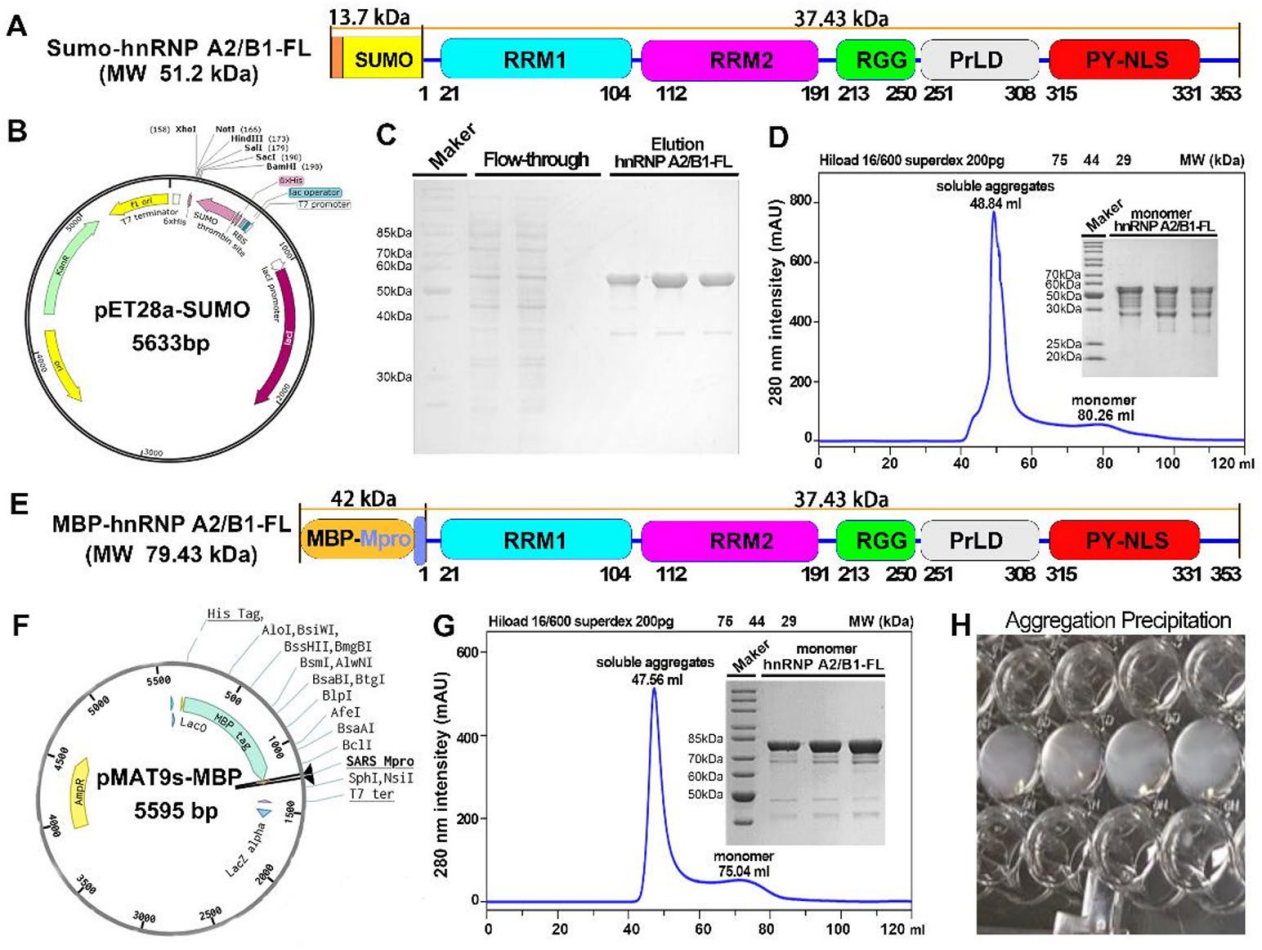
Full-length hnRNP A2/B1 exists as soluble amorphous aggregates in vitro. **(A-B)** N-terminal SUMO-fused full-length hnRNP A2/B1 expression vector features. **(C)** Affinity chromatography purification of N-terminal His-SUMO-fused hnRNP A2/B1, shown by electrophoresis. **(D)** Size-exclusion chromatography analysis of SUMO-tagged hnRNP A2/B1 polymerization state, with purity and molecular weight determined by electrophoresis. **(E-F)** N-terminal MBP-fused full-length hnRNP A2/B1 expression vector characteristics. **(G)** Size-exclusion chromatography results for N-terminal MBP-fused hnRNP A2/B1 polymerization state. (H) Stability test of N-terminal MBP-fused hnRNP A2/B1, indicating aggregation-induced white precipitate formation.

Concentrates were subjected to SEC column (GE Healthcare as S Fig. 1A-D) with mobile phase consisting of 20 mM Tris-HCl (pH 7.5) and 150 mM NaCl. Elution was performed at 1 mL/min under 4 °C to resolve target protein aggregates. Post-separation fractions were analyzed by SDS-PAGE for purity validation and molecular weight determination. ​Different protein molecular weight markers for different size exclusion chromatography.

### SEC-multi-angle light scattering (SEC-MALS)​

An integrated SEC-MALS platform was established using a Wyatt DAWN8 detector coupled to a Superdex 200 Increase 10/300 GL column. The system was equilibrated at 25 °C with buffer containing 20 mM Tris-HCl (pH 7.5), 150 mM NaCl, 5% glycerol, and 0.05% Tween-20. Purified hnRNP A2/B1 (0.5–1.0 mg/mL) was pre-filtered through 0.22 μm PVDF membranes prior to 100 µL injection. Elution was conducted at 0.5 mL/min for 40 min with data acquisition at 1 point/second. Concurrent monitoring of 658 nm laser scattering (8 angles, 15–165°) and differential refractive index (RI) was performed. Molecular weights were calculated via Wyatt ASTRA software using the Berry model.​.

### Isothermal titration calorimetry (ITC)​

Protein (50 µM) and nucleic acid (500 µM) solutions were prepared in 20 mM Tris-HCl (pH 7.5) containing 150 mM NaCl. A 120 µL protein aliquot was loaded into the sample cell of a Malvern ITC200 calorimeter, with nucleic acid solution in the injection syringe. At 25 °C, nucleic acid was injected in 20 equal aliquots, with subsequent injections initiated only after attaining thermodynamic equilibrium. Raw data were analyzed via non-linear least squares fitting (Origin7 software) to determine the dissociation constant (*K*_d_) and thermodynamic parameters of protein-nucleic acid interactions.

### Electrophoretic mobility shift assay (EMSA)​

Seven microliters of 50 µM FAM-labeled nucleic acid solution were transferred to centrifugation tubes. Protein samples (35 µM) were added at volumes of 0, 2, 4, 6, and 8 µL, respectively. Reaction mixtures were adjusted to 15 µL with a buffer containing 20 mM Tris-HCl (pH 8.0) and 100 mM KCl, incubated at room temperature for 30 min, and then mixed with 5 µL of 4× native loading buffer. Samples were separated by 6% native polyacrylamide gel electrophoresis (PAGE) in 0.5×TBE buffer at 0 °C for 20 min. Gel visualization and quantification were performed using the green fluorescence module of the Tanon 5100 imaging system (Bio-Tanon) and its associated image analysis software.​.

### Circular dichroism(CD) spectroscopy analysis

G4-structured ssDNA was synthesized in vitro, and its formation was confirmed by CD spectroscopy via detection of characteristic spectral profiles. Samples (5 µM) were prepared in 20 mM Tris-HCl (pH 8.0) containing 100 mM KCl; 400 µL of each sample was loaded into a 1 cm pathlength cuvette for CD measurements, which were performed at 25 °C with the following parameters: scanning speed 1 nm/0.5 s, response time 0.5 s, and wavelength range 190 ~ 320 nm. Each sample was scanned three times, with resulting spectra averaged, baseline-corrected to subtract buffer background, and smoothed by fitting. Finally, CD spectra (wavelength vs. molar ellipticity) were plotted in GraphPad Prism 8.0, and ssDNA G4 structure formation was verified via characteristic peaks.

### Analytical ultracentrifugation (AUC) analysis

To characterize the oligomeric state of hnRNP A2/B1-DNA complexes, samples were prepared in 20 mM Tris-HCl (pH 8.0), 100 mM NaCl buffer using a Beckman Coulter Optima XL-I ultracentrifuge. 400 µl samples and 400 µl protein buffer (reference) were loaded into sealed sample cells and installed in the rotor.Low-speed centrifugation (5,000 rpm) was performed for vacuum/temperature equilibration and seal integrity checks. The rotor was accelerated to 50,000 rpm, initiating sedimentation coefficient data collection at defined time points. SEDFIT analyzed sedimentation coefficients to calculate solution molecular weights. GraphPad Prism 8.0 plotted sedimentation coefficient distributions against molecular weights.

### Crystallization and data collection

Synthetic nucleic acids, purified by size exclusion chromatography (SEC), were mixed with hnRNP A2/B1 protein at a 1:1.2 molar ratio and incubated for 10 min at room temperature to prepare protein-nucleic acid complexes. Crystallization screening was conducted using the sitting-drop vapor diffusion method with a Gryphon protein crystallization robot. Fifty microliters of screening solution served as the reservoir, and 0.2 µL of crystallization solution was combined with 0.2 µL of the complex sample in each well. Sealed crystallization plates were incubated at 22 °C, and crystal growth was monitored periodically. Crystal quality was optimized by systematically adjusting complex concentration, crystallization solution composition, and drop volume. The latter was increased from an initial 0.4 µL to 4 µL by using plates with larger wells.​.

### Prediction of protein secondary structure​

The SOPMA (Self-Optimized Prediction Method with Alignment) algorithm implemented on the Prabi server (https://npsa-prabi.ibcp.fr/cgi-bin/npsa_automat.pl?p.a. ge=npsa_sopma.html) was employed for secondary structure prediction. Analysis was conducted using default algorithm parameters, including window width and similarity threshold. Probabilities of each residue forming different secondary structure elements were calculated statistically, enabling prediction of the protein’s secondary structure. Visualization maps were generated, presenting the proportions and distributions of α-helices, β-sheets, β-turns, and random coils, providing fundamental data for subsequent higher-order structure analysis.​.

### Prediction of protein 3D structure​ and protein-nucleic acid interaction

Three-dimensional structure prediction of hnRNPA2/B1 was performed using the Uni-Fold platform (https://www.bohrium.com/apps/uni-fold/job?type=app). The full-length amino acid sequence was submitted to the Uni-Fold module on the Bohrium platform with default parameter settings were used for template search to expand the range of homologous structures. In silico molecular modeling was performed using the NPDock web server (https://genesilico.pl/NPDock/), a widely recognized tool specifically designed for predicting RNA-protein and DNA-protein complex structures. This platform integrates four core functional modules: GRAMM-based global macromolecular docking, statistical potential scoring, clustering of top-scored structures, and local refinement, ensuring reliable modeling of protein-nucleic acid binding interfaces. In the present study, 12nt G4-structured ssDNA was designated as the ligand, while truncated RRM-RGG variants served as the receptor for the docking simulation. The 3D models were visualized using Chimera software, which systematically presented spatial conformations, secondary structure distributions, and the positions of key amino acid residues.

## Results​

### Full-length hnRNP A2/B1 exists as soluble amorphous aggregates in vitro​ solution

**Fig. 2:**
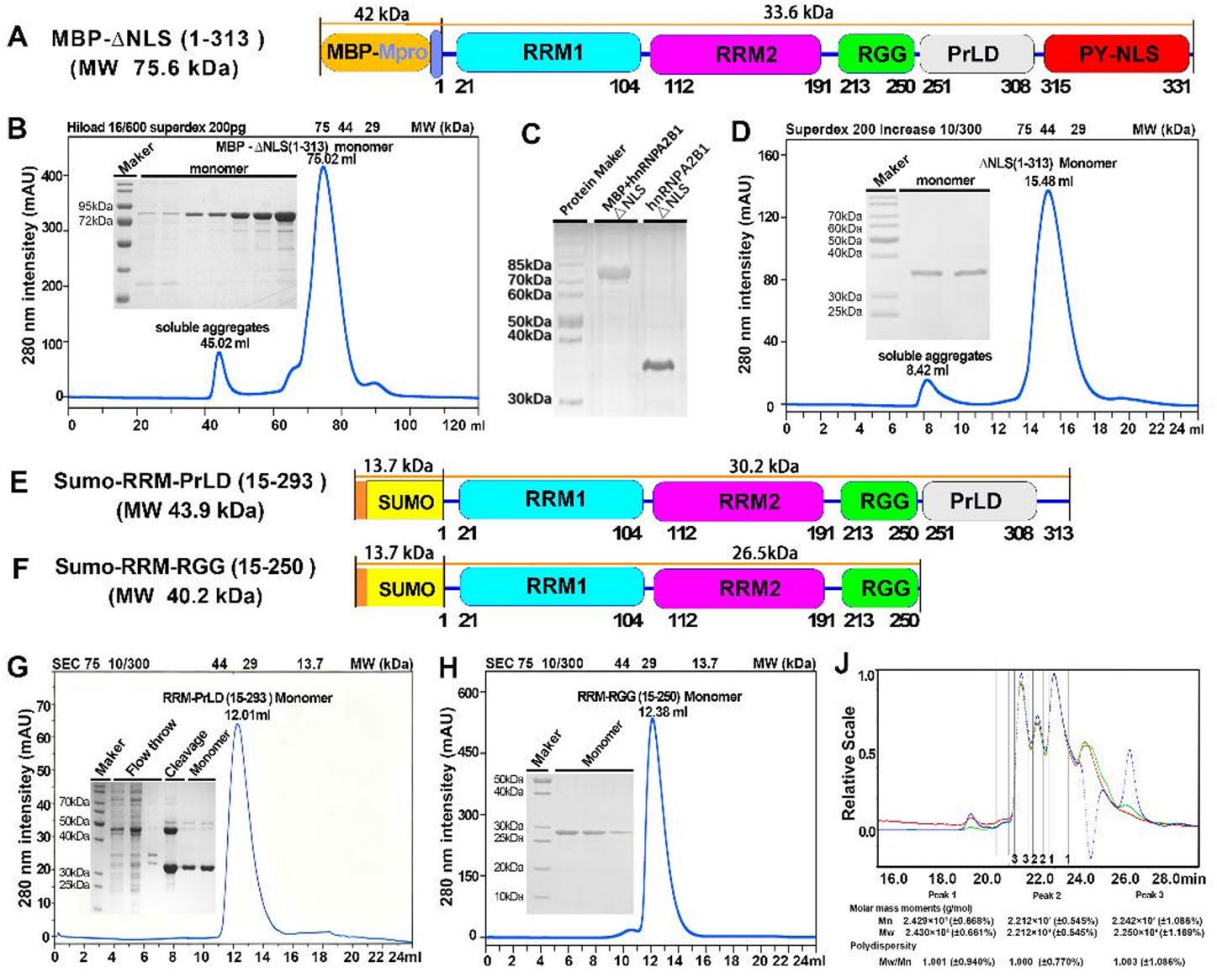
Truncated Variants of hnRNP A2/B1 exist as monomeric state in vitro.​ **(A)** N-terminal MBP-fused ΔNLS (1–313) recombinant vector characterization. **(B)** SEC-based MBP-ΔNLS (1–313) oligomeric state analysis (electrophoretic purity/molecular weight assessment). **(C)** ΔNLS (1–313) electrophoretic profile post-MBP cleavage by SARS-Mpro. **(D)** SEC-characterized ΔNLS (1–313) oligomeric state (purity/molecular weight evaluation). **(E-F)** N-SUMO-tagged truncations RRM-PrLD (15–293) and RRM-RRG (15–250) vector characterization. **(G-H)** SEC and electrophoresis-based characterization of RRM-PrLD (15–293)/RRM-RRG (15–250) oligomeric state, purity, molecular weight. **(J)** SEC-MALLS analysis of RRM-RGG (15–250) oligomeric state.

**Fig. 3:**
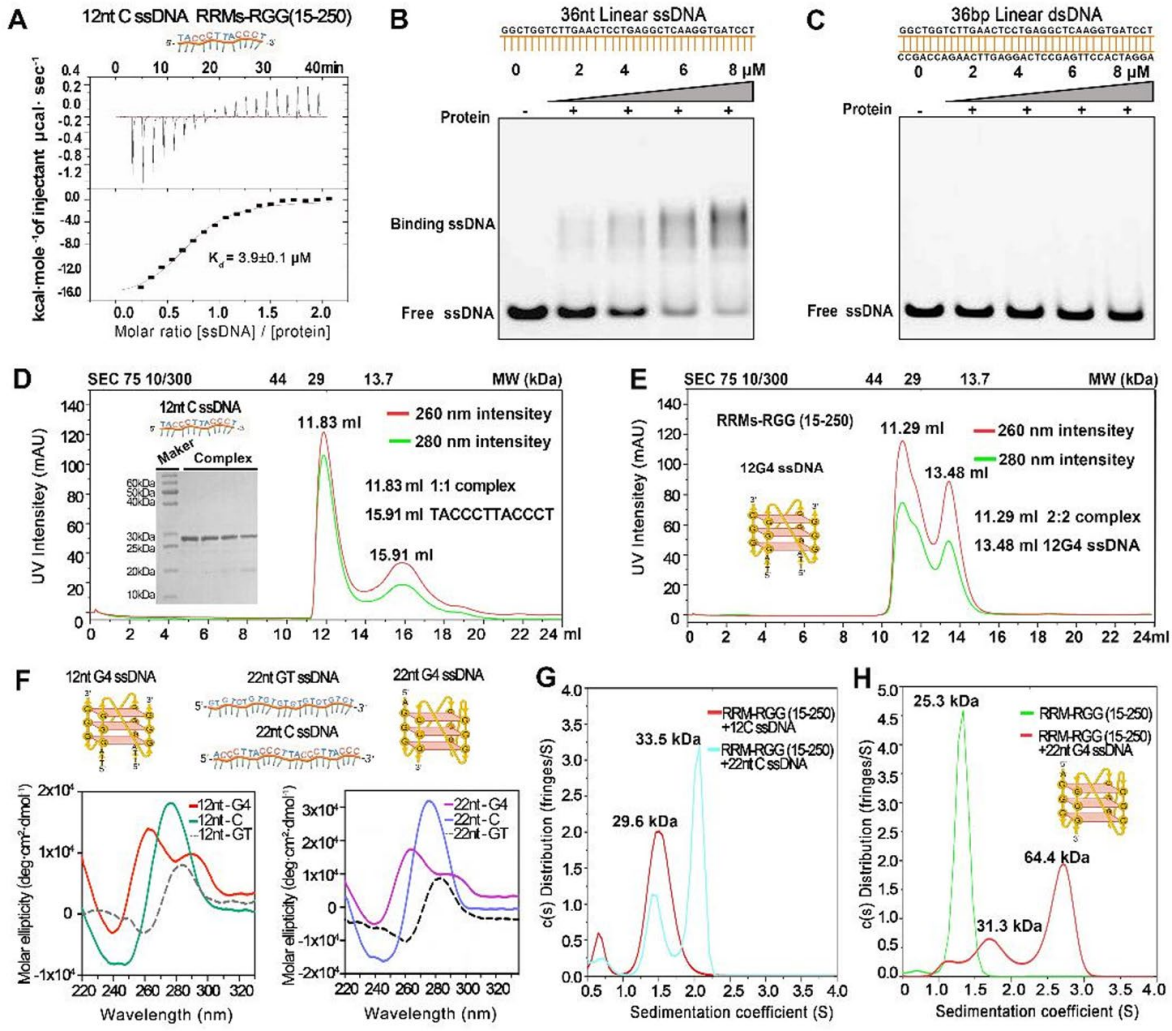
G4 ssDNA binding elicits dimerization of hnRNP A2/B1 truncated variants. **(A)** ITC analysis of the interaction between RRM-RGG (15–250) and 12nt CC-ssDNA. **(B–C)** EMSA-based investigation of the interactions between the RRM-RGG (15–250) truncation and 36nt ssDNA or 36nt dsDNA. **(D–E)** SEC-based oligomeric state analysis of RRM-RGG (15–250) in complex with 12nt CC-ssDNA and 12nt G4-ssDNA, including electrophoretic purity and molecular weight assessment. **(F)** Circular dichroism spectroscopy characterized the structural features of ssDNAs via ellipticity profiles; 12/22nt C ssDNAs are G4-structured mutants. **(G–H)** AUC-based characterization of the oligomeric states of RRM-RGG (15–250) in complex with 12nt CC-ssDNA, 22nt CC-ssDNA, and 22nt G4-ssDNA.

Inspired by our previous research, we designed recombinant expression vectors by integrating an N-terminal Sumo tag with full-length hnRNP A2/B1 (Fig. 1A and B). After purification by affinity chromatography, Sumo-hnRNP A2/B1 showed an estimated purity of 93 ± 1% based on ImageJ densitometry of the target band from SDS-PAGE and consistent with the theoretical value (Fig. 1C, S Fig. 2 and S Table 1). Through SEC characterization of oligomeric states, we discovered that the overwhelming majority (~ 92%) of Sumo-hnRNP A2/B1 eluted at a retention volume of 48.84 ml (Peak area 825 ± 4, 75–44 kDa) (Fig. 1D and S Table 2). In comparison, only about 8% of the sample eluted at the monomeric retention volume of 80.26 ml (Peak area 75 ± 2, 75–44 kDa). This monomeric fraction, with a purity of 41 ± 4%, exhibited multiple degradation bands, suggesting biochemical instability (Fig. 1D, S Fig. 3 and S Table 3).

**Table 1:** Names and sequences of G4-structured and other ssDNAs.

Abbreviations of names	Sequences(5’-3’)
12nt G4-structured ssDNA	TTAGGGTTAGGG
21nt-ssDNA	AGCCAAGGAGCCAGAGAGCAT
22nt G4-structured ssDNA	AGGGTTAGGGTTAGGGTTAGGG
8nt-ssDNA	AGGACAGC
22nt GT control ssDNA	GTGTGTGTGTGTGTGTGTGTGT
12nt C mutant ssDNA	TTACCCTTACCC
22nt C mutant ssDNA	ACCCTTACCCTTACCCTTACCC

**Fig. 4:**
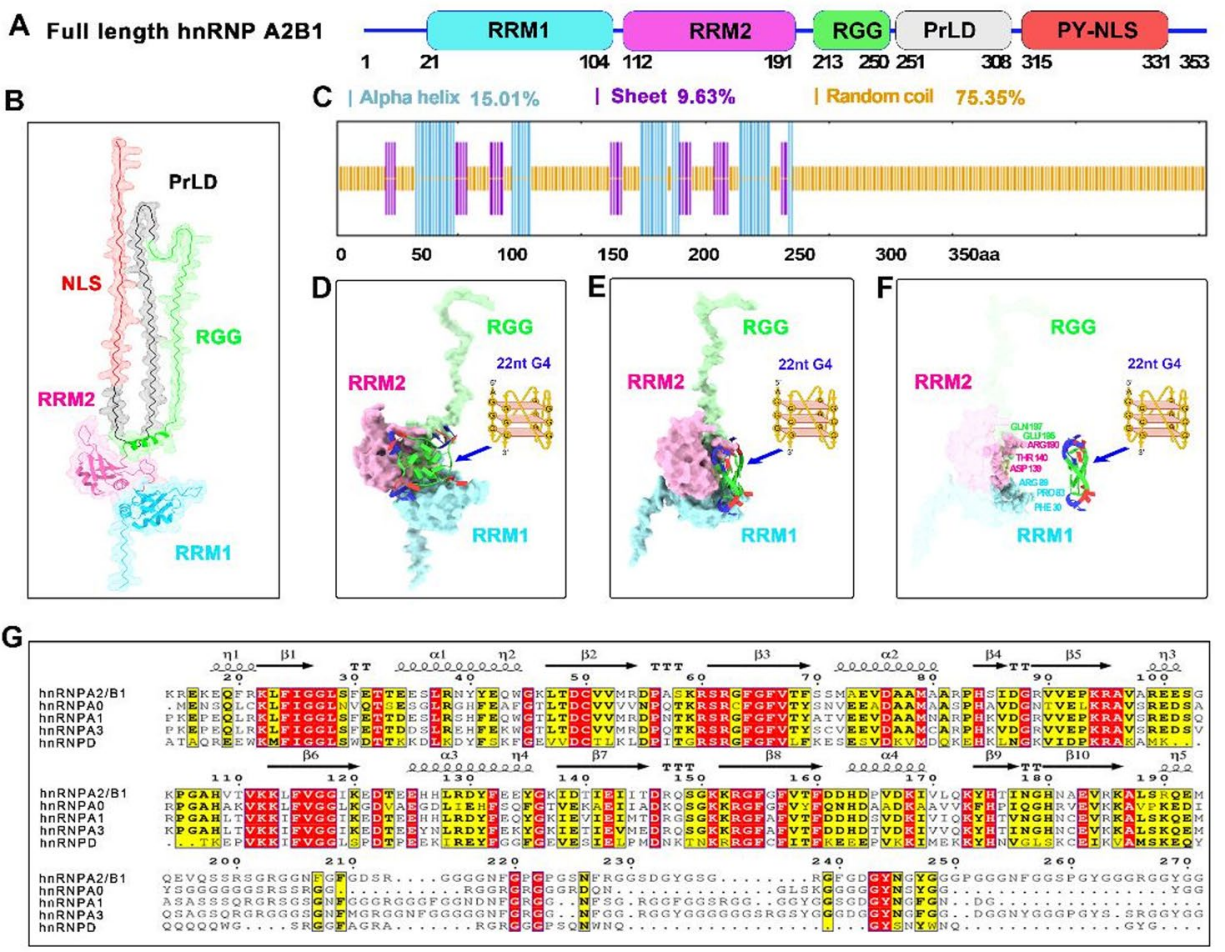
Structural features of hnRNP A2/B1 and key residues mediating its interaction with G4-structured ssDNA. **(A–B)** 3D structure prediction and domain schematic of full-length hnRNP A2/B1 via the Uni-Fold structure prediction platform. **(C)** Secondary structure prediction of full-length hnRNP A2/B1 using the SOPMA web server. **(D–E)** Predicted RRM-RGG (15–250)–12nt G4-ssDNA interaction: structural visualization and 90° rotated view (NPDock web server). RRM1 (blue), RRM2 (pink), RGG (green). **(F)** Key hydrogen bond-mediating residues in the RRM-RGG (15–250)–12nt G4-ssDNA interaction. **(G)** Amino acid sequence and structural alignment of hnRNP A2/B1 with homologous proteins.

To improve the protein’s biochemical characteristics, we developed an expression vector featuring an N-terminal MBP tag fused to hnRNP A2/B1 (Fig. 1E and F). SEC analysis indicated that about 84% of MBP-hnRNP A2/B1 eluted at 47.56 ml (peak area 2891 ± 3, > 1500 kDa), whereas about 17% eluted at 75.04 ml within the monomeric (peak area 571 ± 4,75–44 kDa) range (Fig. 1G and S Table 4). The monomeric fraction demonstrated a high purity of 87 ± 2% and a molecular weight of 80 kDa, consistent with theoretical molecular weight (Fig. 1G, S Fig. 4 and S Table 5). However, upon storage at 4 °C for 24 h, MBP-hnRNP A2/B1 formed noticeable white aggregates (Fig. 1H). Collectively, our oligomeric state analyses strongly indicate that full-length hnRNP A2/B1 predominantly exists as soluble amorphous aggregates—defined as non-crystalline and soluble protein assemblies, exhibiting biochemical properties marked by instability and an amorphous aggregation tendency.

### Truncated variants of hnRNP A2/B1 exist as stable monomers in vitro​

In continuation of our research endeavors, we designed and constructed a recombinant expression vector for the signal peptide-deleted △NLS (1–313) truncated variant of hnRNP A2/B1, featuring an N-terminal MBP tag, based on the protein’s domain characteristics (Fig. 2A). Purification and characterization using a combination of affinity chromatography and SEC revealed that MBP-△NLS (1–313) eluted at an SEC retention volume of 75.02 ml (75–44 kDa), with a purity of ~ 93 ± 0.1% and a molecular weight consistent with the theoretical value of 75.6 kDa (Fig. 2B, S Fig. 5 and S Table 6). After cleavage of MBP tag by SARS-Mpro protease (Fig. 2C and S Fig. 6), the resulting △NLS (1–313) exhibited a high purity 98 ± 1% eluted at an SEC retention volume of 15.48 ml (44–29 kDa), and had a molecular weight matching the theoretical 33.6 kDa, indicating that △NLS (1–313) exists as stable monomer in vitro solution (Fig. 2D, S Fig. 7 and S Table 7).​

To facilitate crystallization and structural determination, we also constructed recombinant vectors for the RRM-PrLD (15–293) and RRM-RGG (15–250) truncated variants, each fused with an N-terminal Sumo tag (Fig. 2E and F). Following purification and characterization with affinity chromatography and SEC, and subsequent removal of the Sumo tag by Ulp1 protease, RRM-PrLD (15–293) eluted with a purity of 85 ± 1% at an SEC retention volume of 12.01 ml (44–29 kDa), suggesting a monomeric state in vitro solution (Fig. 2G, S Fig. 8 and S Table 8). Similarly, the Sumo tag-cleaved RRM-RGG (15–250) eluted at 12.38 ml (29–13.7 kDa), demonstrated a purity of 94 ± 1%, and had a molecular weight of approximately 26 kDa, consistent with the theoretical value, indicating its stable monomeric form in vitro (Fig. 2H, S Fig. 9 and S Table 9). Further confirmation by SEC coupled with multi-angle static light scattering (SEC-MALS) showed that the molecular weight of RRM-RGG (15–250) was approximately 24.29 kDa, close to the theoretical monomeric value, verifying its monomeric state in vitro solution (Fig. 2J).​ This study innovatively demonstrates that the MBP- and Sumo tag-cleaved truncated variants of hnRNP A2/B1, namely △NLS (1–313), RRM-PrLD (15–293), and RRM-RGG (15–250), exist as homogeneous monomers in vitro solution. These findings provide a structural basis for subsequent crystallization screening and elucidation of the nucleic acid recognition mechanism of hnRNP A2/B1, and lay a theoretical foundation for in vitro targeted drug screening.

### G4-structured ssDNA undergoes dimerization of hnRNP A2/B1 truncated variants

To comprehensively elucidate the DNA-binding properties and oligomeric states of hnRNP A2/B1 truncated variants △NLS (1–313) and RRM-RGG (15–250), isothermal titration calorimetry (ITC) was employed as a quantitative analytical approach. We utilized ITC to dissect the interaction dynamics between the RRM-RGG (15–250) truncation and 12nt C ssDNA (Fig. 3A). The resulting dissociation constant value *K*_d_ of 3.9 ± 0.1 µM, not only underscores the high-affinity interaction but also firmly establishes the strong binding properties of this protein domain towards 12nt C-ssDNA. EMSA was used to assess the double-stranded DNA (dsDNA) binding ability of RRM-RGG (15–250). The gray value of the 36nt ssDNA and RRM-RGG (15–250) complex band was 12,660 ± 862, which was significantly high than that of the 36nt dsDNA complex band (171 ± 65, *P* < 0.05) group of the same sequence (Fig. 3B-C, S Figs. 10 and 11 and Table S10). This result indicates that RRM-RGG (15–250) binds ssDNA but not dsDNA.

We further investigated the impact of 12nt G4-structured ssDNA on the oligomeric states of △NLS (1–313) and RRM-RGG (15–250). SEC analysis showed that the △NLS (1–313) and 12nt G4 ssDNA complex eluted at a retention volume of 14.52 ml (75–44 kDa), whereas free △NLS (1–313) and 12nt G4 ssDNA eluted at 16.02 ml and 17.34 ml (both ~ 29 kDa), respectively (S Fig. 12). These results imply that 12nt G4 ssDNA may drive △NLS (1–313) dimerization.​ The RRM-RGG (15–250) and 12nt G4 ssDNA complex eluted at 11.29 mL (44–29 kDa), while complex of non G4-structured 12nt CC ssDNA eluted at 11.83 mL (~ 29 kDa) retention (Fig. 3D and S Fig. 13), suggesting that 12nt G4 ssDNA may induce RRM-RGG (15–250) homodimerization (Fig. 3E).

​ To confirm the G4 structural characteristics of the ssDNAs prepared in vitro, Circular Dichroism (CD) spectroscopy was employed to detect their characteristic ellipticity profiles. The CD spectrum results of the 12nt G4-structured ssDNA exhibited distinct signature peaks of G4 structure formation at 240 nm, 260 nm, and 290 nm (Fig. 3F). In contrast, no such characteristic G4 structure peaks were observed in the CD spectra of the 12nt C ssDNA (12nt G4-structured ssDNA mutant) or the control 12nt GT ssDNA. Similarly, the 22nt G4-structure ssDNA displayed prominent G4 structure ellipticity peaks at 230 nm, 250 nm, and 290 nm in its CD spectrum. Conversely, the CD spectra of the 22nt C ssDNA (22nt G4-structure ssDNA mutant) and the control 12nt GT ssDNA lacked these diagnostic G4 structure peaks (Fig. 3F; Table 1). Consistent with previous reports, our experimental results confirm that both the 12nt and 22nt G4-structured ssDNAs successfully form G4 structures—motifs abundant in the genomes of diverse viruses^27,28^. AUC further validated the oligomeric states of these complexes. The molecular weight of the RRM-RGG (15–250) and 22nt G4 ssDNA complex was approximately 64.4 kDa, compared to 25.3 kDa for pure RRM-RGG (15–250), indicating that 22nt G4 ssDNA can induce RRM-RGG (15–250) homodimerization (Fig. 3H). In contrast, the molecular weights of complexes formed by non-G4 22nt CC ssDNA and 12nt C ssDNA with RRM-RGG (15–250) were 29.6 kDa and 33.5 kDa, suggesting that non-G4 ssDNA fails to drive RRM-RGG (15–250) dimerization (Fig. 3G). ​ More significantly, they undergo homodimerization upon interaction with ssDNA forming G4 structures. These findings provide critical experimental evidence for elucidating the molecular mechanisms by which hnRNP A2/B1 regulates its oligomeric states to exert antiviral and other diverse functions.

### Crystallization proves difficult for hnRNPA2B1 truncated variants and nucleic acid complexes

To elucidate the structural basis of hnRNP A2/B1’s antiviral functions, we systematically subjected two purified truncated variants, RRM-RGG (15–250) and △NLS (1–313), to a series of crystallization screening conditions. Employing the sitting-drop vapor diffusion method in 96-well plates for the initial screening, we identified three putative protein crystals (S Fig. 14 A and B). However, no protein crystal diffraction data could be acquired, indicating that the putative crystals were, in fact, salt crystals.

To investigate the structural interactions between hnRNP A2/B1 truncated variants and nucleic acids, we synthesized five nucleic acid molecules with diverse lengths and sequences: 12nt G4-ssDNA, 21nt ssDNA, 22nt G4-ssDNA, and 8nt-ssRNA (Table 1). These synthesized oligonucleotides were subsequently incubated with the two purified truncated variants, resulting in the formation of eight well characterized complexes. Through crystallization screening of each complex, five potential protein-nucleic acid complex crystals were tentatively identified (S Fig. 14 C and D). Despite these efforts, no diffraction data were obtained for these complexes, indicating that the grown crystals were also salt crystals. In spite of conducting exhaustive crystallization screening on hnRNP A2/B1 truncated variants and their complexes with ssDNA molecules of diverse lengths and sequences, no suitable crystals for X-ray diffraction could be obtained. These findings imply that these truncated variants likely adopt flexible, multi-conformational, and disordered structures, which not only impede crystallization but also present major challenges for crystallographic structure determination.

### Structural features of hnRNP A2/B1 and key residues mediating its interaction with G4-structured ssDNA

To investigate the structural features underlying the aggregation propensity of full-length hnRNP A2/B1 and the crystallization difficulties of its ΔNLS (1–313) and RRM-RGG (15–250) truncations in complex with nucleic acids, we performed three-dimensional structure predictions of the full-length protein using Uni-Fold with confidence scores (predicted local distance difference test, pLDDT) for each residue. The predicted model reveals that hnRNP A2/B1 consists of an N-terminal disordered region (residues 1–14; pLDDT values 50 ~ 70), followed by two central RRM domains—RRM1 (residues 15–97, containing 2 α-helices and 6 β-strands; pLDDT > 90) and RRM2 (residues 112–188, with 2 α-helices and 5 β-strands; pLDDT > 90)—an RGG (residues 190–250; pLDDT ≤ 50) formed by a single α-helix, and a C-terminal region dominated by extensive random coil (Figs. 4B). The C-terminal PrLD displayed moderate pLDDT values ≤ 50, which is typical for prion-like domains (residues 252–313) with context-dependent flexibility. To validate the predicted structural features, we performed secondary structure analysis. The overall composition was quantified as 15.01% α-helix, 9.63% β-strand, and 75.35% random coil (Fig. 4C and S Fig. 15). An arginine-glycine-glycine-rich domain (RGG, residues 190–250) predicted to be predominantly intrinsically disordered, with the SOPMA algorithm suggesting a potential α-helix segment (residues 210–230) that is not supported by Uni-Fold 3D modeling. To dissect the molecular basis of the interaction between hnRNP A2/B1-derived truncated RRM-RGG variants and G4-structured ssDNA, we performed in silico molecular modeling via the NPDock platform—a widely used tool for predicting protein-nucleic acid binding interfaces—using 12nt G4-structured ssDNA as the ligand and truncated RRM-RGG variants as the receptor. This computational analysis predicted that specific evolutionarily non-conserved residues form potential hydrogen-bonding networks with the G4-structured ssDNA (Fig. 4D and E). Specifically, non-conserved key residues mediating hydrogen bonding were identified across domains: PHE30, ARG82, and ARG89 in RRM1; ASP139, THR140, and ARG190 in the adjacent RRM2; and GLU195/GLN197 in the C-terminal RGG domain, which contribute to the stability of the G4-structured ssDNA complex via hydrogen bonding (Fig. 4F and G). Collectively, these structural predictions suggest that hnRNP A2/B1 has a domain-differentiated architecture: the N-terminus, C-terminal PrLD, and NLS domain are intrinsically disordered, potentially driving its aggregation and poor crystallization upon nucleic acid binding. NPDock simulations show that the RRM-RGG truncate stabilizes complexes with 12nt G4-ssDNA via hydrogen bonds mediated by non-conserved key residues in the RRM1, RRM2, and RGG domains.

## Discussion

In the present investigation, biophysiological and structural factures attributes of full length of hnRNP A2/B1 and truncates, with emphasis on defining their oligomeric states and G4 structured ssDNA induced homodimerization. To surmount challenges associated with low expression levels and poor solubility, a strategic approach involving fusion tag technology was implemented. The small ubiquitin-like modifier (Sumo) tag, renowned for enhancing protein solubility by promoting proper folding, was employed to augment expression yields and mitigate aggregation. This tag can be selectively removed by Ulp1 protease, enabling recovery of native proteins^29,30^. Additionally, the maltose-binding protein (MBP) tag was utilized to enhance protein stability during expression, followed by affinity purification and cleavage with SARS-Mpro protease to obtain homogeneous, high-purity samples^31,32^. However, during the subsequent purification processes, the protein manifested characteristics consistent with soluble amorphous aggregates in solution, accompanied by notable degradation and the formation of white precipitates.

To conduct a systematic investigation into the critical factors governing the polymerization state of hnRNP A2/B1, three truncated variants with sequential deletions of the C-terminal domains were designed and prepared: △NLS (1–313), RRM-PrLD (15–293), and RRM-RGG (15–250). Biophysical characterization data demonstrated that all three truncated variants existed stably as monomers in solution. This finding led to the inference that the C-terminal region encompassing the NLS domain (residues 314–341) may contribute to the aggregation propensity of full-length hnRNP A2/B1. Consistent with this observation, NLS deletion abrogates hnRNP K degradation, highlighting a potentially conserved regulatory role of the NLS in hnRNP stability modulation^33^. Notably, full-length hnRNP A2/B1 aggregation is unlikely to be solely NLS-mediated, as the C-terminal PrLD and N-terminal disordered region also contribute to structural flexibility and protein aggregation (Fig. 4B and C).

Minor molecular weight discrepancies of RRM-RGG (15–250) (AUC: 25.3 kDa; SEC-MALS: 24.29 kDa) stem from buffer composition differences and intrinsic RGG domain flexibility. The AUC buffer was glycerol- and Tween-20-free, while the SEC-MALS buffer contained 5% glycerol and 0.05% Tween-20—additives that alter the protein’s hydrodynamic radius and refractive index. SEC-MALS quantifies apparent molecular weight via the Stokes radius, while AUC measures the native sedimentation coefficient. These factors may contribute to the observed discrepancies. Despite these variations, both techniques confirm that RRM-RGG (15–250) is monomeric, validating our findings.

Integrating the results of multiple unsuccessful crystallization screening attempts for complexes with different lengths and sequences of ssDNAs, it is hypothesized that the hnRNP A2/B1 truncated variants may possess highly dynamic structures hinder the ordered assembly and crystallization of protein-nucleic acid complexes. Tertiary structure prediction of the full-length hnRNP A2/B1 using the Uni-Fold algorithm demonstrated that both the N- and C-terminal regions adopt intrinsically disordered conformations. SOPMA secondary structure prediction further revealed that intrinsically disordered structures account for up to 75% of the protein sequence, providing additional support for this hypothesis. Uni-Fold/SOPMA prediction discrepancies may stem from SOPMA’s sequence alignment/statistical rule reliance, while Uni-Fold’s deep learning-based inter-residue distance constraints enhance disordered domain prediction robustness^34^.

Despite prior studies showing hnRNP RRM-RGG domains bind G4-nucleic acids, our SEC/AUC data demonstrate that hnRNP A2/B1 RRM-RGG (15–250) undergoes homodimerization upon G4-ssDNA binding—unlike hnRNP A1 and hnRNP D, which form 1:1 nucleic acid complex^35–38^. NPDock simulations demonstrated that G4-structured ssDNA binds and stabilizes the RRM-RGG (15–250) truncation via non-conserved key residues distinct from those of other hnRNP family members. These findings indicate hnRNP A2/B1 isoform-specific oligomeric regulatory mechanisms. Nevertheless, future work should employ hnRNP A2/B1 knockout cells and HSV-1 G4 mutant viruses to verify whether G4 binding and homodimerization are required for the protein’s antiviral immune function.

The crystal structure of the 12nt G4-ssDNA/RRM-RGG (15–250) complex was not obtained despite employing the crystallization platform reported in previous work^16^. our work demonstrates that integrated biophysical approaches can yield mechanistic insights even in the absence of crystal structures—particularly for conformationally flexible proteins that are recalcitrant to crystallization. To dissect the molecular basis of hnRNP A2/B1 RRM-RGG truncate-G4-ssDNA interaction, we performed in silico modeling via NPDock (a standard protein-nucleic acid binding prediction tool) using 12nt G4-ssDNA as ligand and RRM-RGG truncates as receptor (Figs. 4D-F). This analysis predicted specific non-conserved residues form potential hydrogen-bonding networks with G4-ssDNA (Figs. 4G). However, these predictions require validation via cryo-EM structural determination, which remains a future research priority. Thus, the current study provides phenotypic evidence of G4-specific binding rather than a complete molecular recognition mechanism. The current study was conducted exclusively in vitro, and the physiological relevance of G4-structured ssDNA induced dimerization in antiviral immunity remains to be experimentally corroborated. Future investigations should employ viral G4-structured DNA motif mutants and hnRNP A2/B1 knockout cell lines to validate whether G4-structured DNA binding and dimerization are indispensable for TBK1-IRF3 pathway activation^15^. Additionally, the role of full-length hnRNP A2/B1’s aggregation in antiviral signaling—distinct from the monomeric or dimeric states of the truncated variants utilized in this study—merits systematic investigation.

Our study defines the oligomeric states of the full-length hnRNP A2/B1 form (soluble amorphous aggregates) and its truncated variants (stable monomers), and validates G4-ssDNA-induced homodimerization in RRM-RGG domain-containing variants. These systematic characterizations reported herein not only clarify the molecular basis of hnRNP A2/B1 oligomeric regulation but also establish a robust experimental foundation for the subsequent in vitro screening of hnRNP A2/B1-specific inhibitors and the rational design of antiviral or disease-modifying therapeutics.

## Conclusions

Our study directly addresses three core knowledge gaps in hnRNP A2/B1 biology: (1) Defining the oligomeric states of full-length (soluble amorphous aggregates) and truncated (stable monomers) forms (2) Identifying the C-terminal NLS and PrLD domains as key mediator of aggregation (3) Validating G4-ssDNA induction of homodimerization in RRM-RGG-containing variants. Most notably, SEC and AUC confirmed that hnRNP A2/B1 RRM-RGG (15–250) truncated variant undergoes homodimerization induced by G4-enriched viral ssDNA, which is abundant in the genomes of diverse viruses and regulates viral replication. This G4-ssDNA-dependent dimerization suggests a potential physiological role for this process in hnRNP A2/B1’s antiviral response, as the protein binds viral G4 motifs and undergoes dimerization—a key step in it signaling activation. However, in vivo validation (e.g., viral infection models with G4 motif mutants) is required to confirm this mechanism. Collectively, our findings clarify three core aspects of hnRNP A2/B1 biology: the oligomeric states of full-length and truncated forms, the ssDNA-binding selectivity of the RRM-RGG (15–250) truncated variant, and the role of intrinsically disordered regions in mediating protein instability. More importantly, our data uncover a critical link between hnRNP A2/B1’s oligomeric state and its function as a nuclear DNA sensor, providing a biophysical framework to guide future studies on hnRNP A2/B1-mediated antiviral responses and the rational design of targeted inhibitors.

## Supplementary Information

Below is the link to the electronic supplementary material.

Supplementary Material 1

## Data Availability

Data is provided within the manuscript or supplementary information files.
