# Supplementary material for: Molecular recognition and induced dimerization of hnRNP A2/B1 truncations by G-quadruplex single strand DNA
Source: Sci Rep. 2026 Mar 26;16:10970. doi: 10.1038/s41598-026-44646-7 (PMC13039485; doi:10.1038/s41598-026-44646-7)
Supplement: Supplementary file 1 — Supplementary Material 1 [file 41598_2026_44646_MOESM1_ESM.doc]

**Supplemental Data for**

**Molecular Recognition and Induced Dimerization of hnRNP A2/B1 Truncations by G-Quadruplex single strand DNA**

Dilidaer Shahatibieke**^1^**, Xiaohui Tang**^1^**, Xuanfang Zheng**^1^**, Yue Liu**^4^**, Abudoureyimu Abula **^1,2,3*^**

**1** Department of microbiology, School of Basic Medical Sciences, Xinjiang Medical University, Urumqi, China

**2** Xinjiang Key Laboratory of Molecular Biology for Endemic Diseases

**3** State Key Laboratory of Pathogenesis, Prevention, Treatment of Central Asian High Incidence Diseases, Xinjiang Medical University, Urumqi, China

**4** The State Key Laboratory of Pharmaceutical Biotechnology, School of Life Sciences, Nanjing University, Nanjing, China.

*** Corresponding authors:** Abudoureyimu Abula ([dg1730001@smail.nju.edu.cn](mailto:dg1730001@smail.nju.edu.cn))

**Funding:** This work was supported by the Natural Science Foundation of Xinjiang Uygur Autonomous Region (Grant No. 2022D01C441), Youth Program of National Natural Science Foundation of China (Grant No. 32201032)

15 figures, 11 tables

**S Table 1. Sumo-hnRNPA2/B1 purity analysis for Fig.1C**

| Target protein band gray value | Contaminating protein band gray value | Percentage (target protein band relative to the total gray value) |
| --- | --- | --- |
| 9264.953 | 826.87 | 91.80% |
| 14609.08 | 867.042 | 94.39% |
| 11352.18 | 720.213 | 94.03% |
| Mean±SD | | 93±1% |

**S Table 2. Sumo-hnRNPA2/B1 oligomeric state for Fig.1D**

| Retention volume of peak | Soluble inclusions peak area value （Percentage） | Monomeric peak area value（Percentage） |
| --- | --- | --- |
| Peak area | 9264.953（91.81%）  9263.345（91.82%）  9247.783（91.83%） | 826.870（8.91%）  824.961（8.18%）  822.567（8.17%） |
| Area Mean±SD | 9258.7±9.5 | 824.8±2.2 |
| Percentage Mean±SD | 92±0.01%（～92%） | 8±0.01%（about 8%） |

**S Table 3. Sumo-hnRNPA2/B1 purity analysis for Fig.**1D

| Target protein band gray value | Contaminating protein band gray value | Percentage (target protein band relative to the total gray value) |
| --- | --- | --- |
| 14400.418 | 17049.886 | 45.79% |
| 12053.368 | 18758.836 | 39.12% |
| 11378.711 | 17857.693 | 38.92% |
| Mean±SD | | 41±4% |

**S Table 4. MBP-hnRNPA2/B1 oligomeric state for Fig.**1G

| Retention volume of peak | Soluble inclusions peak area value （Percentage） | Monomeric peak area value（Percentage） |
| --- | --- | --- |
| Peak area | 2887.736（83.51%）  2893.325（83.60%）  2890.436（83.42%） | 570.403（16.49%）  567.432（16.40%）  574.342（16.58%） |
| Area Mean±SD | 2890.5±2.8 | 570.7±3.5 |
| Percentage Mean±SD | 84±0.09%（about 84%） | 17±0.1%（about 17%） |

**S Table 5. MBP-hnRNPA2/B1purity analysis for Fig.1G**

| Target protein band gray value | Contaminating protein band gray value | Percentage (target protein band relative to the total gray value) |
| --- | --- | --- |
| 8791.811 | 1528.359 | 85.19% |
| 11688.054 | 1471.054 | 88.82% |
| 16978.974 | 2413.309 | 87.56% |
| Mean±SD | | 87±2% |

**S Table 6. MBP-△NLS (1-313) purity analysis for Fig.2B**

| Target protein band gray value | Contaminating protein band gray value | Percentage (target protein band relative to the total gray value) |
| --- | --- | --- |
| 5974.418 | 476.607 | 92.61% |
| 5971.672 | 470.987 | 92.69% |
| 5890.882 | 473.231 | 92.56% |
| Mean±SD | | 93±0.1% |

**S Table 7. △NLS (1-313) purity analysis for Fig.2**D

| Target protein band gray value | Contaminating protein band gray value | Percentage (target protein band relative to the total gray value) |
| --- | --- | --- |
| 9912.589 | 167.778 | 98.34% |
| 11485.711 | 429.142 | 96.40% |
| 9883.761 | 138.071 | 98.62% |
| Mean±SD | | 98±1% |

**S Table 8.** RRM-PrLD（15-293 ）purity analysis for Fig.2G

| Target protein band gray value | Contaminating protein band gray value | Percentage (target protein band relative to the total gray value) |
| --- | --- | --- |
| 14466.25 | 2573.832 | 84.90% |
| 18521.08 | 3066.054 | 85.80% |
| 14181.3 | 2446.418 | 85.29% |
| Mean±SD | | 85±1% |

**S Table 9. RRM-RGG（15-250 ）purity analysis for Fig.2H**

| Target protein band gray value | Contaminating protein band gray value | Percentage (target protein band relative to the total gray value) |
| --- | --- | --- |
| 15318.56 | 918.749 | 94.34% |
| 11723.95 | 866.87 | 93.12% |
| 5510.397 | 403.728 | 93.17% |
| Mean±SD | | 94±1% |

**S Table 10. EMSA binding analysis for Fig.3B and 3C**

| Target binding band gray  value for Fig.2B | | Target binding band gray  value for Fig.2C |
| --- | --- | --- |
|  | 4358.426 | 120.243 |
|  | 7390.054 | 166.536 |
|  | 15306.258 | 264.364 |
|  | 23583.957 | 132.828 |
| Mean±SD | 12659.67 ± 862.02 | 171±65 |
| Independent Samples t-test | | t = 2.90（df=6）*p* < 0.05 |

**
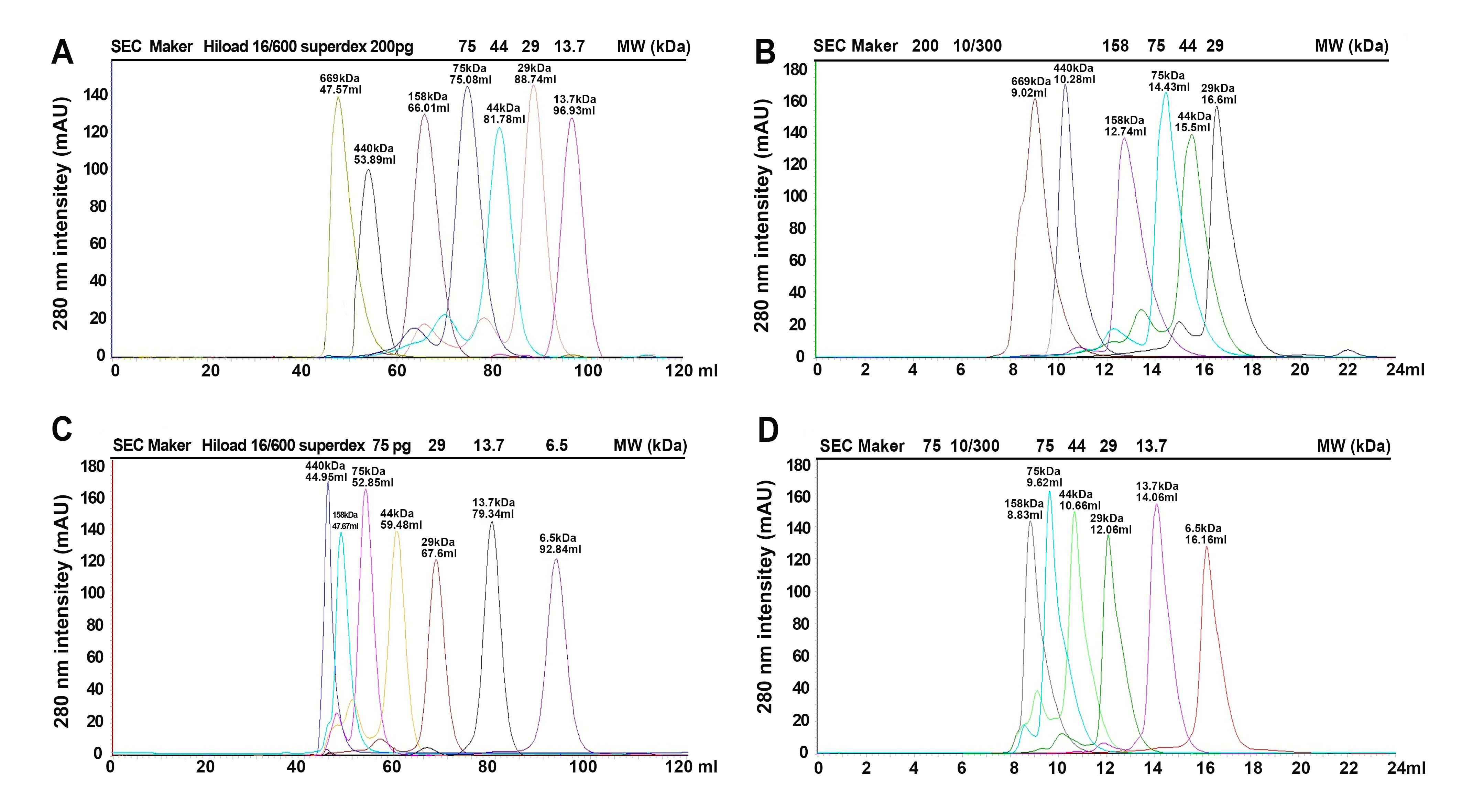
**

**S Figure 1. Different protein molecular weight markers for different size exclusion chromatography (SEC).** Protein molecular weight markers including thyroglobulin (670,000 Da), bovine γ-globulin (158,000 Da), and chicken ovalbumin (44,000 Da), which cover a broad range of molecular weights.


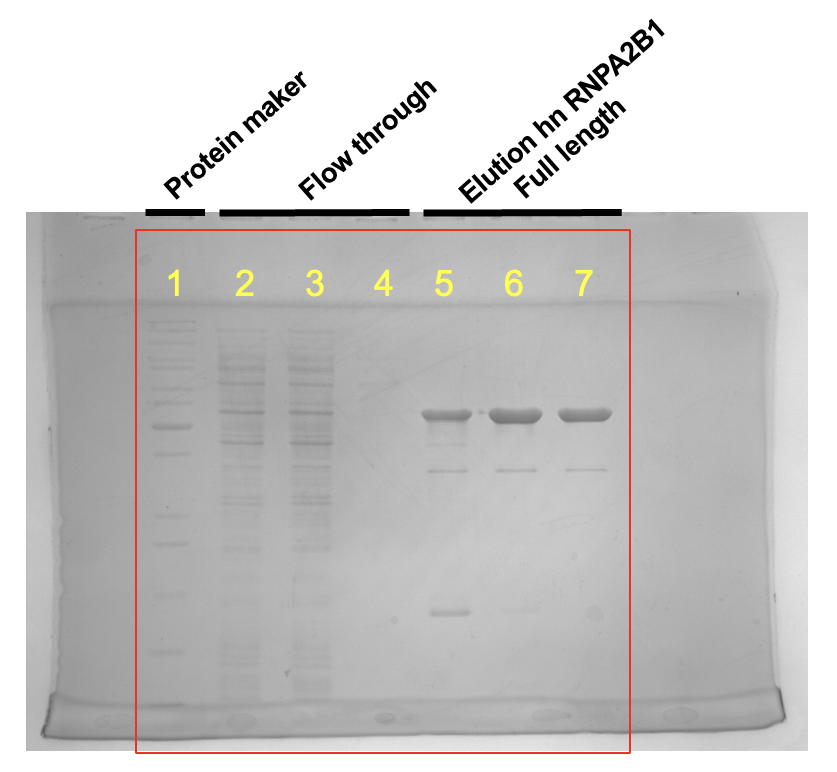


**S Figure 2. Affinity chromatography purification of N-terminal His-SUMO-fused hnRNP A2/B1, shown by electrophoresis for Figure 1C.**  Lane 1: protein marker; Lane 2-4: Affinity chromatography flow through purity; Lane 5-7: SUMO-hnRNPA2B1-FL purity and molecular weight.


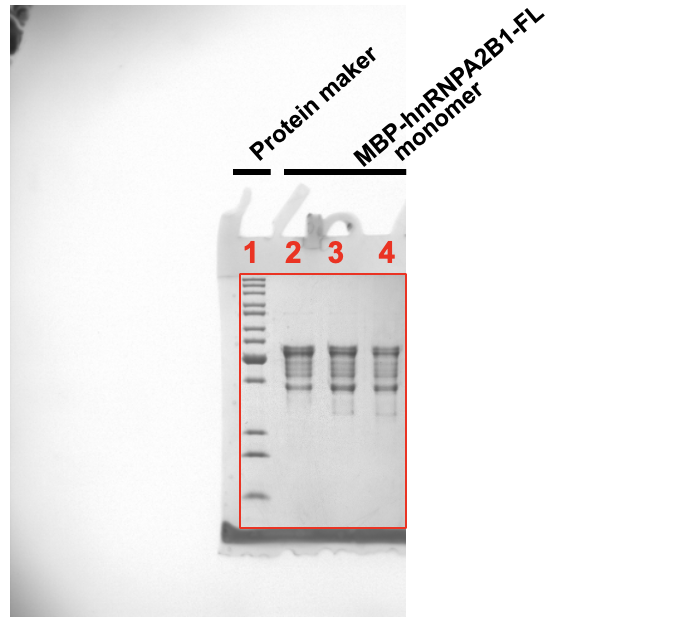


**S Figure 3. Size-exclusion chromatography analysis of SUMO-tagged hnRNP A2/B1 polymerization state, with purity and molecular weight determined by electrophoresis for Figure 1D.** Lane 1: Protein molecular weight marker; Lanes 2–4: Representative SEC chromatography fractions of SUMO-tagged hnRNP A2/B1, demonstrating protein degradation and molecular weight.


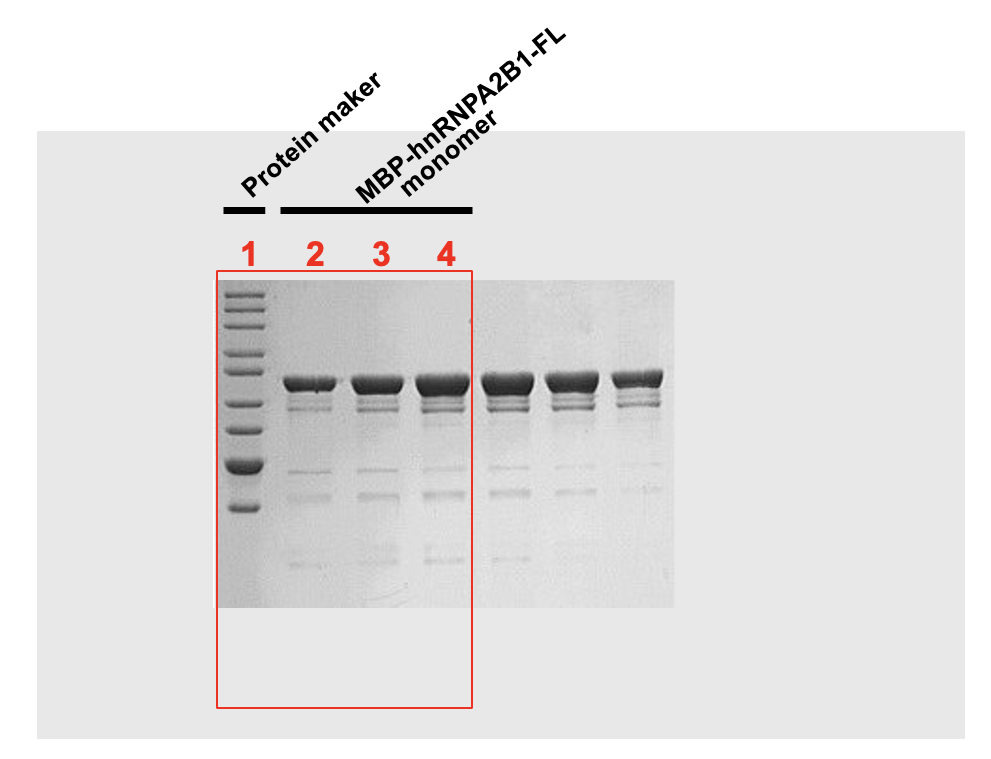


**Figure 1G. Affinity chromatography purification of N-terminal His-SUMO-fused hnRNP A2/B1, shown by electrophoresis.** Lane 1: protein marker; Lane 2-4: Affinity chromatography flow through purity; Lane 5-7: SUMO-hnRNPA2B1-FL purity and molecular weight.

**S Figure 4. Size-exclusion chromatography results for N-terminal MBP-fused hnRNP A2/B1 polymerization state, with purity and molecular weight determined by electrophoresis for Figure 1G.** Lane 1: Protein molecular weight marker; Lanes 2–4: Representative SEC chromatography fractions of N-terminal MBP-fused hnRNP A2/B1, demonstrating protein degradation and molecular weight.


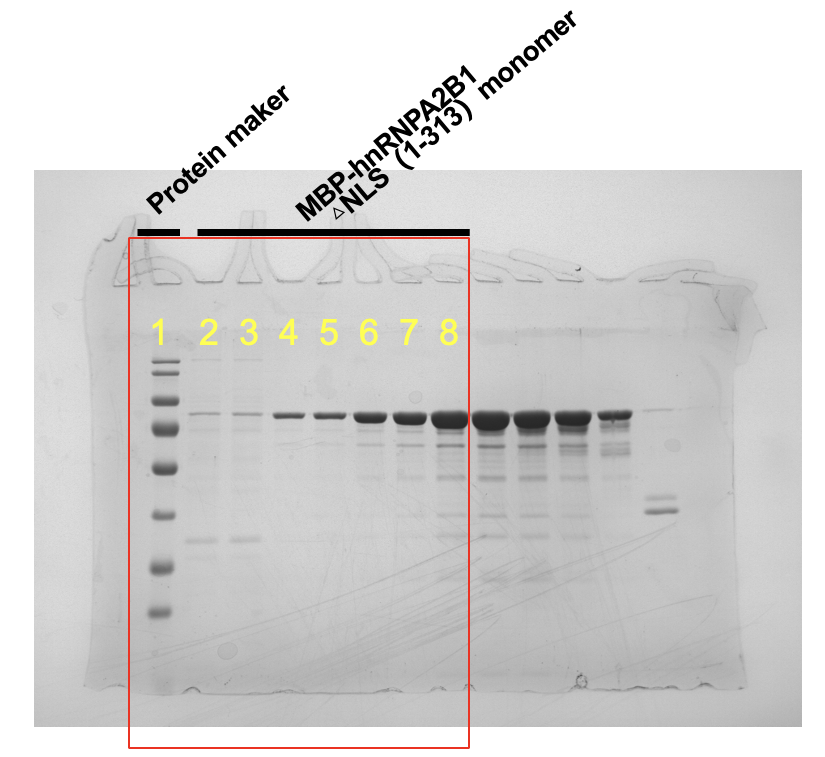


**S Figure 5. Affinity chromatography purification of N-terminal His-SUMO-fused hnRNP A2/B1, shown by electrophoresis for Figure 2B.** Lane 1: Protein molecular weight marker; Lanes 2–8: Representative SEC chromatography fractions of MBP-hnRNPA2B1-ΔNLS (1–313), demonstrating protein purity and molecular weight.

**
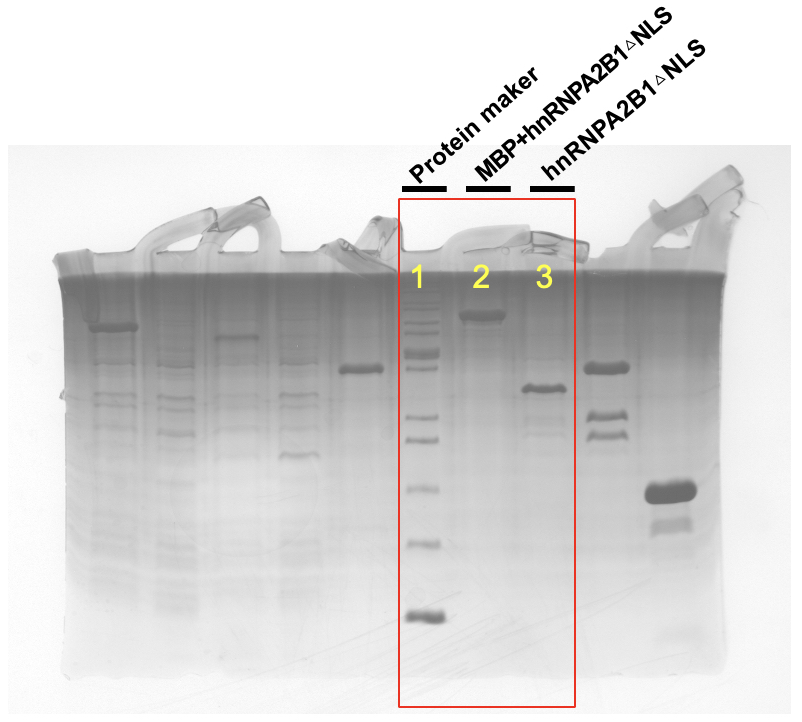
**

**S Figure 6. Electrophoretic analysis of ΔNLS (1–313) following MBP cleavage by SARS-Mpro for Figure 2C.** Lane 1 (left): protein marker; Lane 2: MBP-hnRNPA2B1ΔNLS fusion protein; Lane 3: purified hnRNPA2B1ΔNLS after MBP removal.

**
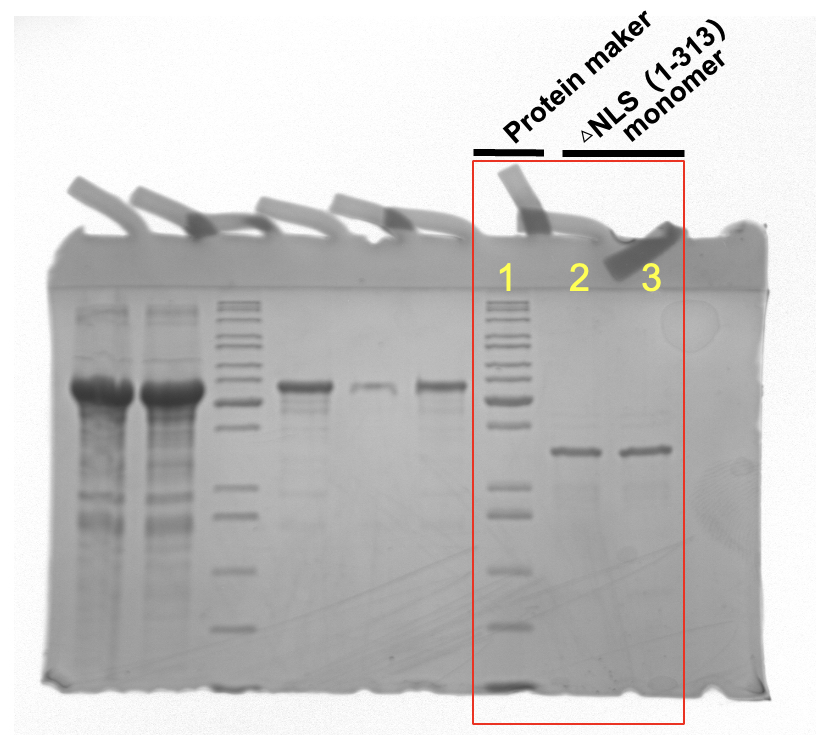
**

**S Figure 7. SEC-characterized ΔNLS (1-313) oligomeric state purity and molecular weight evaluation for Figure 2D.** Lane 1 (left): protein marker; Lane 2 and Lane 3: purified hnRNPA2B1ΔNLS (1-313) after MBP removal.


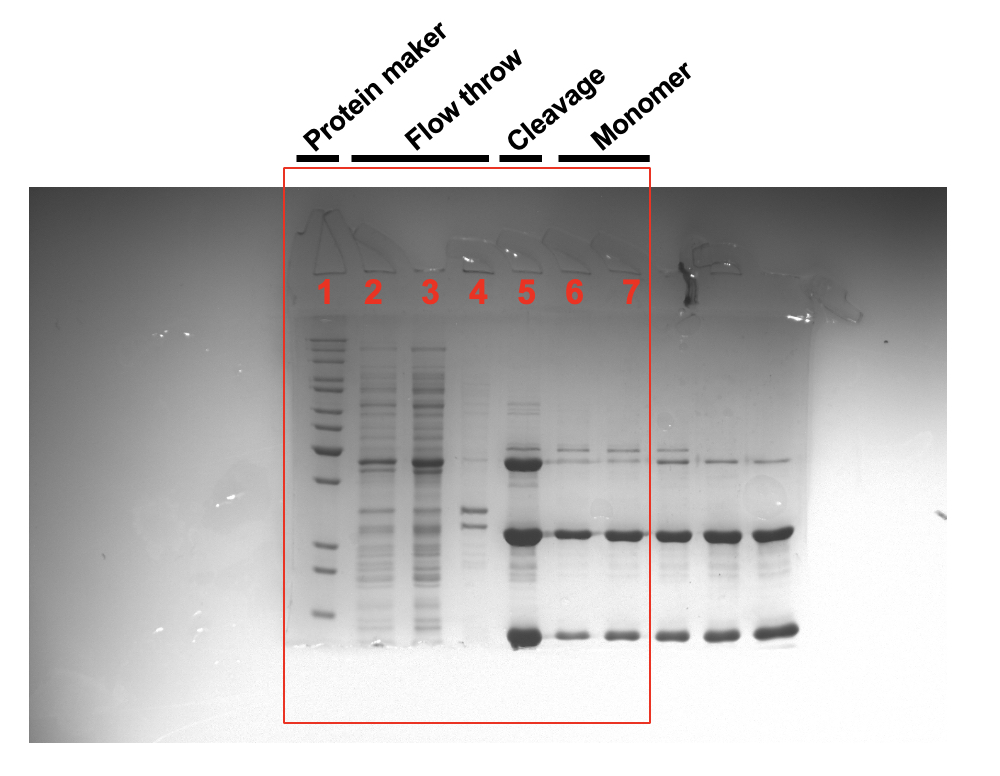


**S Figure 8. SEC and electrophoresis-based characterization of RRM-PrLD (15-293) oligomeric state purity and molecular weight for Figure 2G.** Lane 1: Protein marker. Lanes 2–4: Flow-through fractions after Ulp1 cleavage of the N‑terminal SUMO tag from RRM‑PrLD (15–293). Lane 5: SUMO cleavage reaction mixture. Lanes 6–7: Purified RRM‑PrLD (15–293) following SUMO removal, analyzed by SEC for oligomeric state and molecular weight.


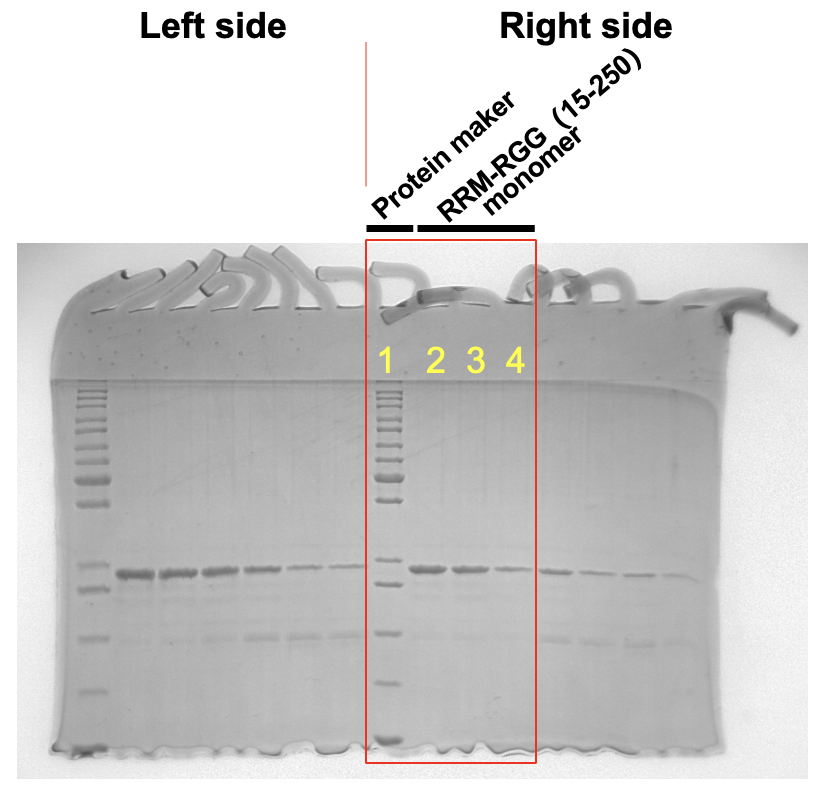


**S Figure 9. SEC and electrophoresis-based characterization of RRM-RRG (15-250) oligomeric state purity and molecular weight for Figure 2H.** Lane 1: protein marker; Lane 2-4: hnRNPA2B1 RRM-RRG (15-250) oligomeric state purity and molecular weight.


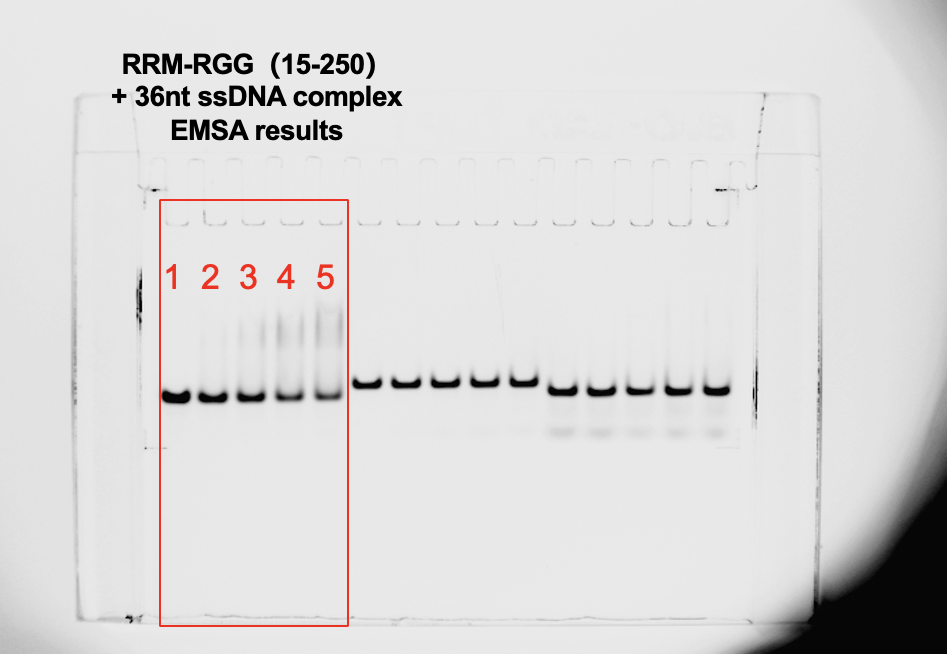


**S Figure 10. EMSA-based investigation of the interactions between the RRM-RGG (15–250) truncation and 36nt ssDNA for Figure 3B.** Lane 1: Control reaction in the absence of RRM-RGG (15–250) protein, showing the migration of 36 nt single-stranded (ss) DNA. Lanes 2–8: Electrophoretic mobility shift assays (EMSAs) of RRM-RGG (15–250) protein at increasing concentrations (2, 4, 6, 8 µM) incubated with 36 nt ss DNA, showing the formation of protein–nucleic acid complexes.


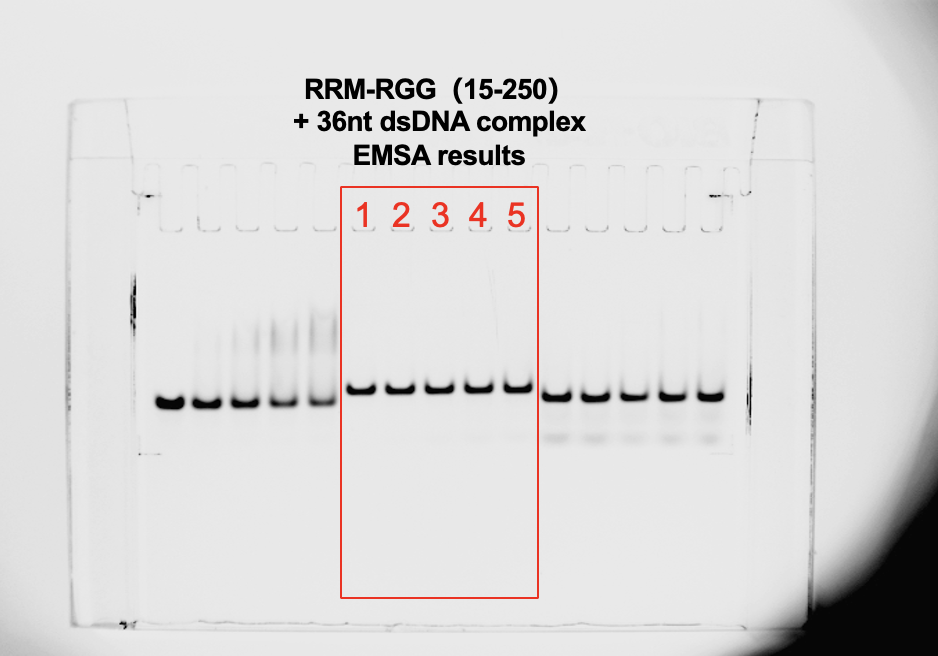


**S Figure 11. EMSA-based investigation of the interactions between the RRM-RGG (15–250) truncation and 36nt dsDNA for Figure 3C.** Lane 1: Control reaction in the absence of RRM-RGG (15–250) protein, showing the migration of 36nt double-stranded (ds) DNA. Lanes 2–8: Electrophoretic mobility shift assays (EMSAs) of RRM-RGG (15–250) protein at increasing concentrations (2, 4, 6, 8 µM) incubated with 36nt dsDNA, showing the formation of protein–nucleic acid complexes.

**
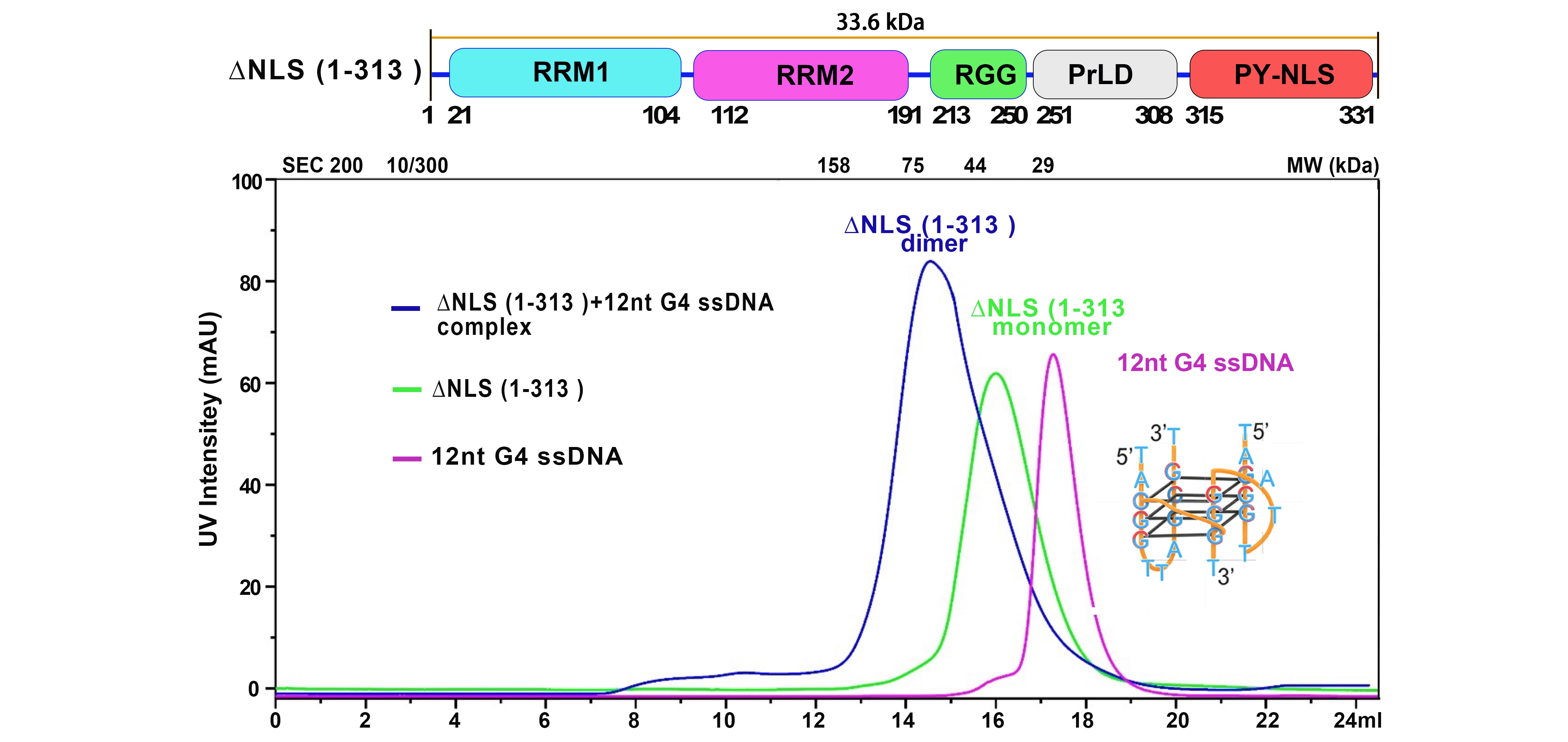
**

**S Figure 12. Specific ssDNA binding elicits dimerization of hnRNP A2/B1 truncated variants.** The SEC-based oligomeric state analysis of △NLS (1-313) complexes with 12nt G4-ssDNA and pure △NLS (1-313) protein and 12nt G4-ssDNA.


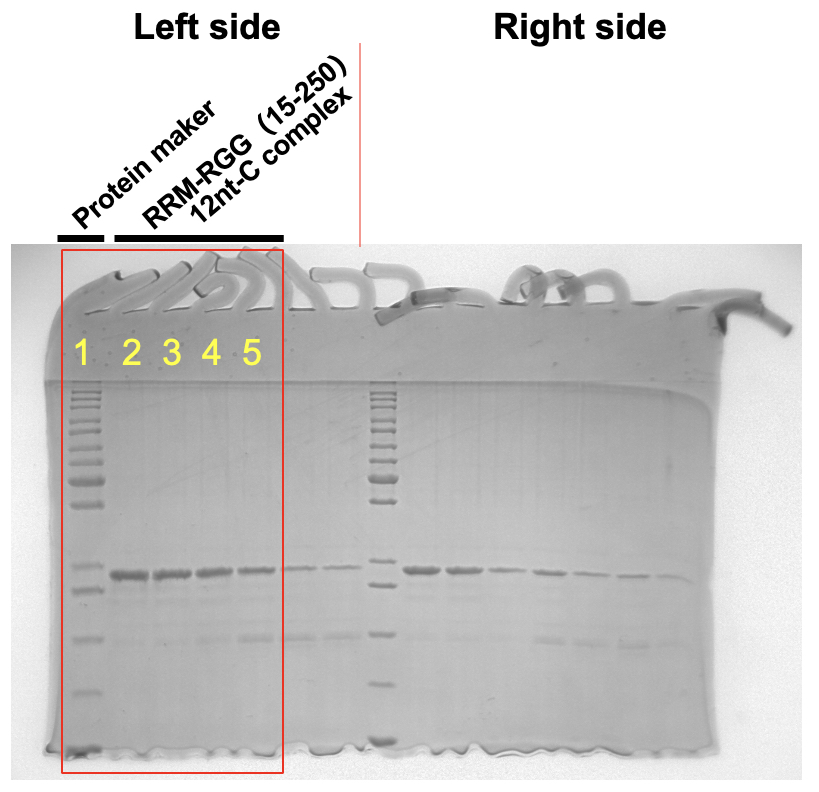


**S Figure 13. SEC and electrophoresis-based characterization of RRM-RRG (15-250) oligomeric state purity and molecular weight for Figure 3D.** Lane 1: protein marker; Lane 2-4: hnRNPA2B1 RRM-RRG (15-250) oligomeric state purity and molecular weight.

**
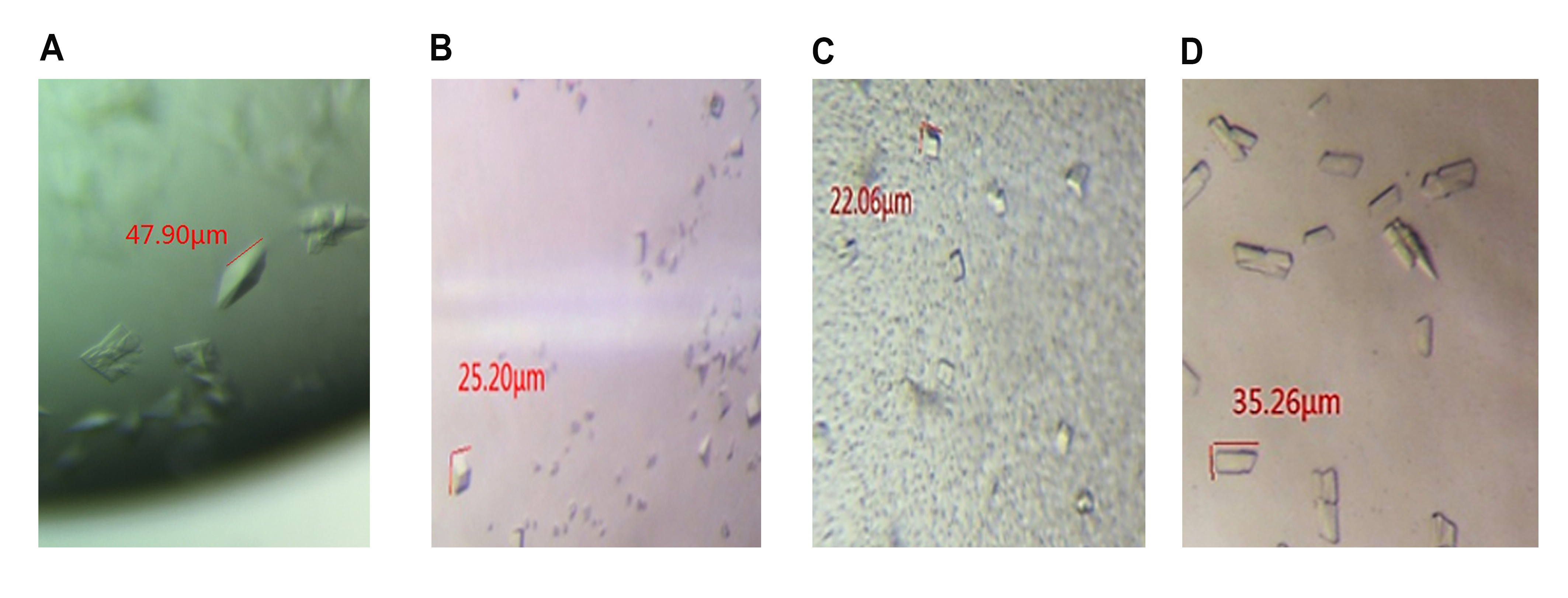
**

**S Figure 14. Crystallization Proves Difficult for hnRNPA2B1 Truncated Variants and Nucleic Acid Complexes.** (A) Crystals of the RRM-RGG (15–250)/12nt G4-ssDNA complex were obtained in 200 mM NaCl, 10% PEG 8000, pH 7.2, with dimensions of ~47.9 μm. (B) In 150 mM MgCl₂, 5% glycerol, pH 6.5, the complex formed crystals measuring ~25.2 μm. (C) Crystallization of the RRM-RGG (15–250)/22nt G4-ssDNA hybrid complex yielded crystals ~22.06 μm in size. (D) Conversely, crystallization of the RRM-RGG (15–250)/8nt-ssDNA hybrid complex.

**
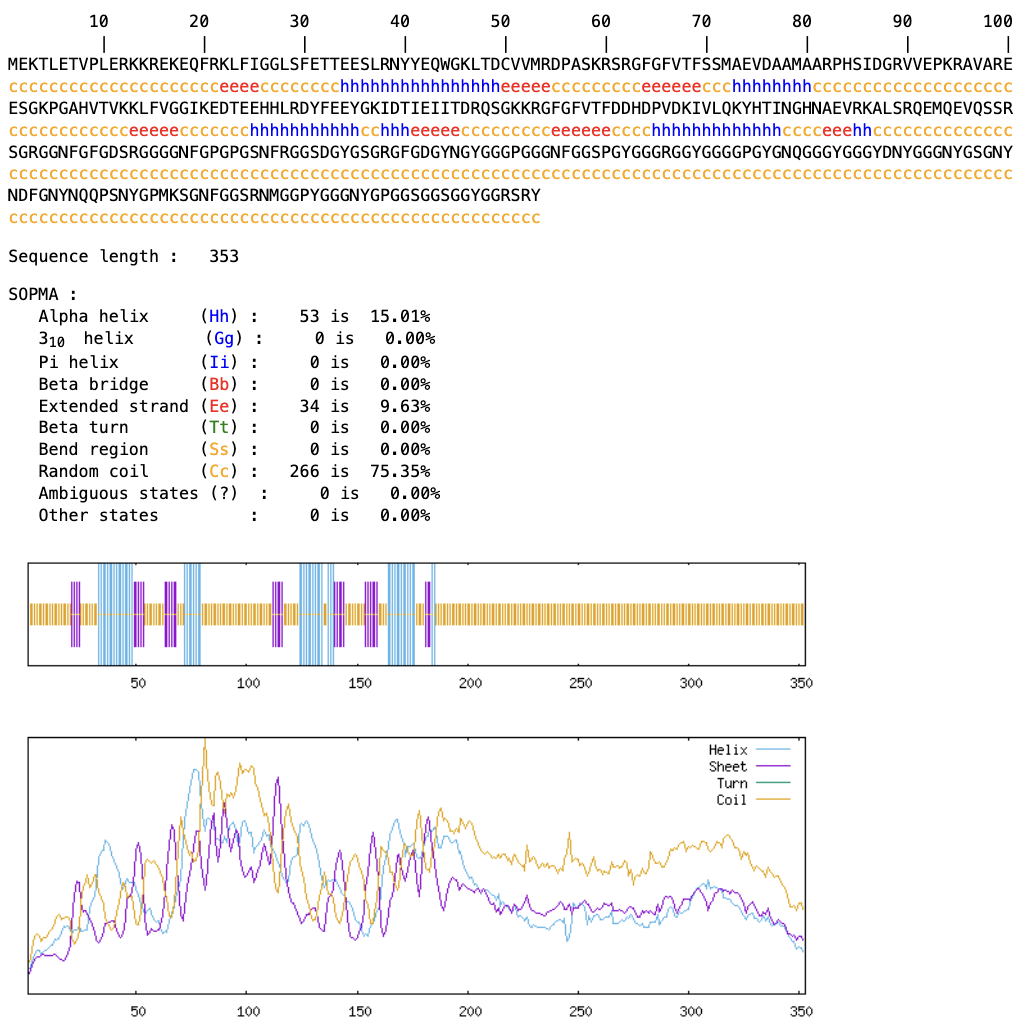
**

**S Figure 15.** **The C-Terminal Disordered Region Implicated in Crystallization Hindrance of hnRNP A2/B1.** The SOPMA (Self-Optimized Prediction Method with Alignment) algorithm implemented on the Prabi server was employed for secondary structure prediction.
